# Supplementary material for: Inhibition of SFTSV replication in humanized mice by a subcutaneously administered anti-PD1 nanobody
Source: EMBO Mol Med. 2024 Feb 16;16(3):8. doi: 10.1038/s44321-024-00026-0 (PMC10940662; doi:10.1038/s44321-024-00026-0)
Supplement: Supplementary file 1 — Table EV1 [file 44321_2024_26_MOESM1_ESM.docx]

**Table EV1. The table summary of the binding of NbP45 with PD-1 protein tested by BLI.**

| Conc.(nM) | Response | KD (M) | Ka (1/Ms) | Kd (1/s) | RMax | Full R^2 |
| --- | --- | --- | --- | --- | --- | --- |
| 300.00 | 0.1424 | 8.64E-09 | 3.62E+05 | 3.13E-03 | 0.1555 | 0.0057 |
| 100.00 | 0.0955 | 1.29E-08 | 4.61E+05 | 5.95E-03 | 0.1146 | 0.0111 |
| 33.33 | 0.0476 | 1.85E-08 | 6.01E+05 | 1.11E-02 | 0.0643 | 0.0078 |
| 11.11 | 0.0331 | 1.57E-08 | 7.76E+05 | 1.22E-02 | 0.0464 | 0.0056 |
| 3.70 | 0.0243 | 1.30E-08 | 8.04E+05 | 1.05E-02 | 0.0331 | 0.0027 |
| 1.23 | 0.012 | 8.39E-09 | 5.86E+05 | 4.91E-03 | 0.0154 | 0.0008 |
