# Supplementary material for: Inhibition of SFTSV replication in humanized mice by a subcutaneously administered anti-PD1 nanobody
Source: EMBO Mol Med. 2024 Feb 16;16(3):8. doi: 10.1038/s44321-024-00026-0 (PMC10940662; doi:10.1038/s44321-024-00026-0)
Supplement: Supplementary file 2 — Appendix [file 44321_2024_26_MOESM2_ESM.pdf]

Appendix Figures

Inhibition of SFTSV replication in humanized mice by a subcutaneously administered anti-PD1 nanobody

Authors: Mengmeng Ji[1†], Jiaqian Hu[2†], Doudou Zhang[2], Bilian Huang[2], Shijie Xu[2,3], Na Jiang[2], Yuxin Chen[4*], Yujiong Wang[1*], Xilin Wu[2,6*] and Zhiwei Wu[1,2,5,6*]

[*]**Correspondence:** wzhw@nju.edu.cn

Table of Contents

Appendix Figure S1. Apoptosis of the T lymphocytes during SFTSV infected PBMC: Page 2

Appendix Figure S2. Evaluation of NbP45 therapeutic efficacy in SFTSV-infected NCG-HuPBL mice: Page 3

Appendix Figure S3. Characterization of NbP45: Page 4

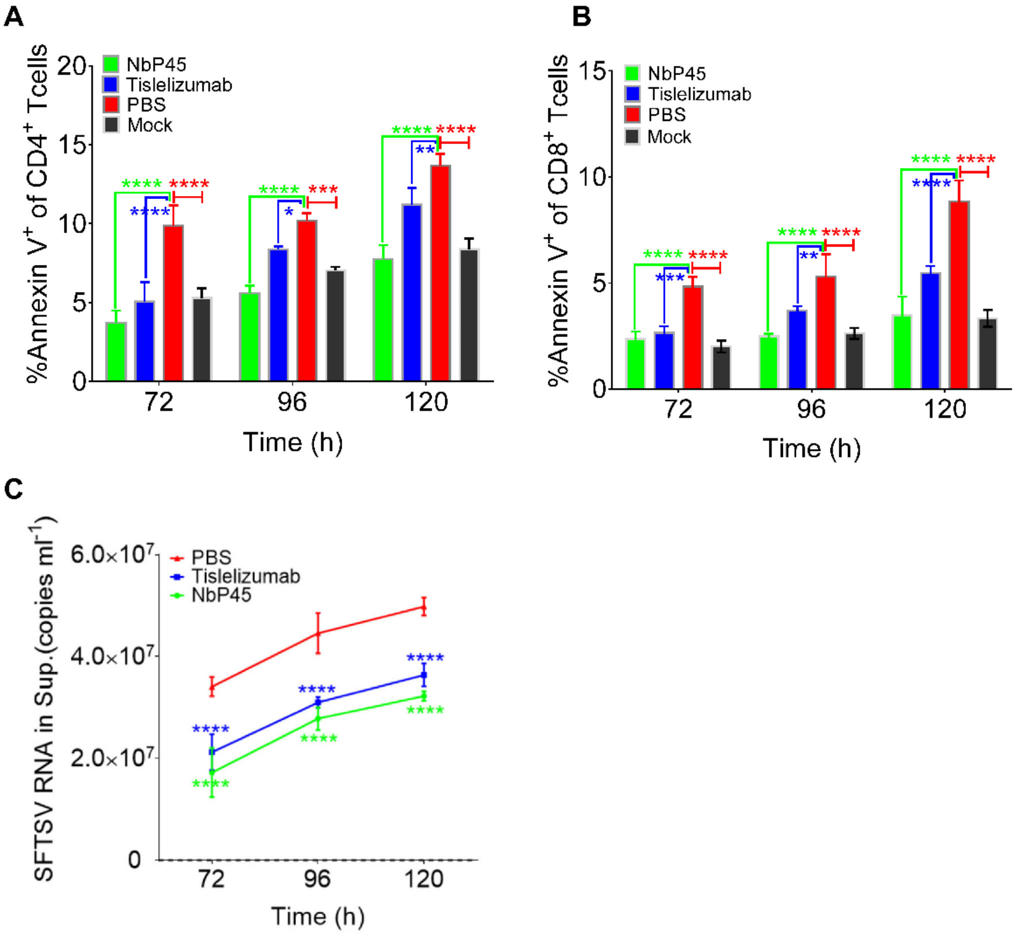

Appendix Figure S1. Apoptosis of the T lymphocytes during SFTSV infected PBMC.

A, B. Annexin V^+ expression of $CD4^+$ (A) and $CD8^+$ (B) T cell was summarized for the uninfected (*n* = 3), SFTSV (MOI = 1) infected (*n* = 3) and treated controls (*n* = 3) at 72/96/120 h.

C. The inhibitory activity of NbP45 (*n* = 3) or Tislelizumab (*n* = 3) against SFTSV (MOI = 1) infection of PBMCs at 72/96/120 h. Each line represents data from a group with indicated treatment.

Data are shown as mean ± SEM. Two-way ANOVA with Tukey's test was performed to compare SFTSV infection, treatment group with control group. *$p<0.05$; **$p<0.01$; ***$p<0.001$; ****$p<0.0001$.

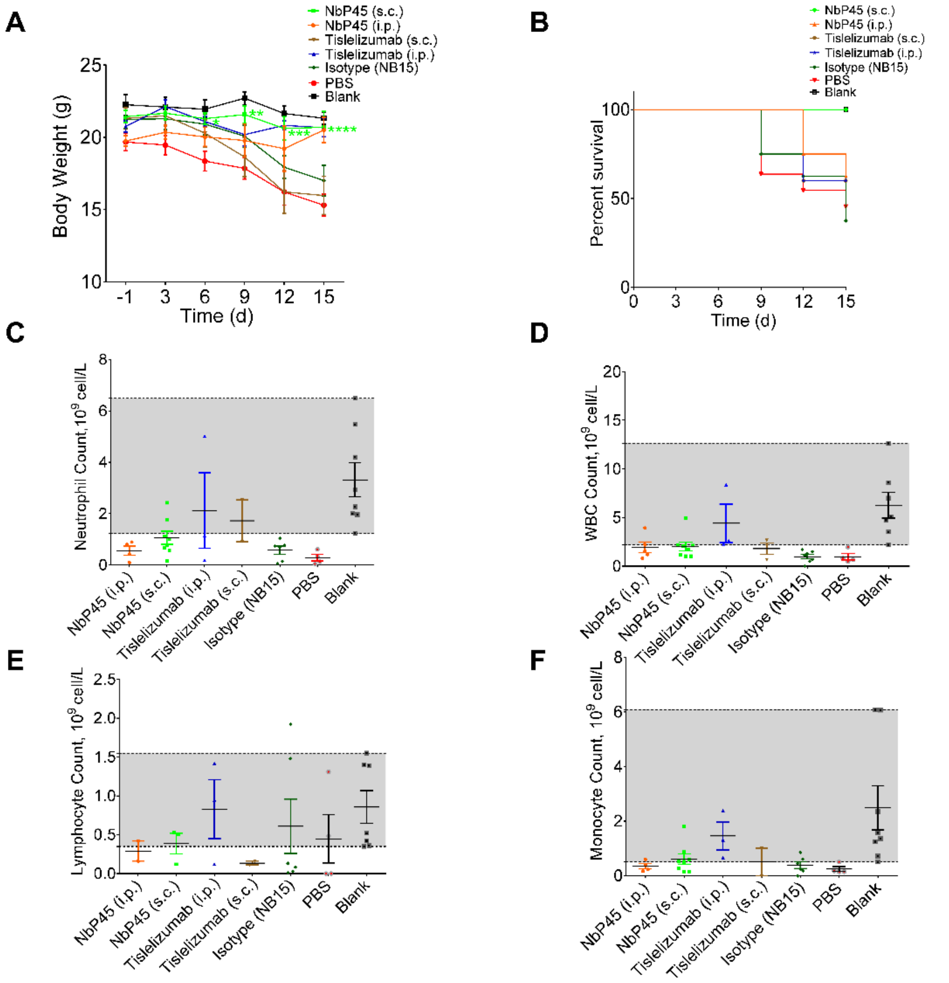

Appendix Figure S2. Evaluation of NbP45 therapeutic efficacy in SFTSV-infected NCG-HuPBL mice.

A, B. Relative weight (A) and survival (B) among 7 groups of color-coded NCG-HuPBL mice including no SFTSV challenge (n = 8), SFTSV challenge with PBS treatment as control (n = 11), SFTSV challenge with isotype (NB15) treatment as control (n = 8), NbP45 treatment by s.c. (n = 8) or i.p. (n = 8), Tislelizumab treatment by s.c. (n = 5) or i.p. (n = 5). Each line represents data from 1 group.

C-F. WBCs (C), neutrophils (D), lymphocytes (E), and monocytes (F) were analyzed in 7 groups of NCG-HuPBL mice infected with SFTSV at 15 days including no SFTSV challenge (n = 8), SFTSV challenge with PBS treatment as control (n = 5), SFTSV challenge with isotype (NB15) treatment as control (n = 7), NbP45 treatment by s.c. (n = 8) or i.p. (n = 5), Tislelizumab treatment by s.c. (n = 3) or i.p. (n = 3). The normal range is between the 2 dashed lines. Each dot represents data from 1 mouse.

Data are shown as mean ± SEM. Two-way ANOVA with Tukey's test was performed to compare treatment group with control group (PBS). *ns*, no significance; $*p<0.05$; $**p<0.01$; $***p<0.001$; $****p<0.0001$.

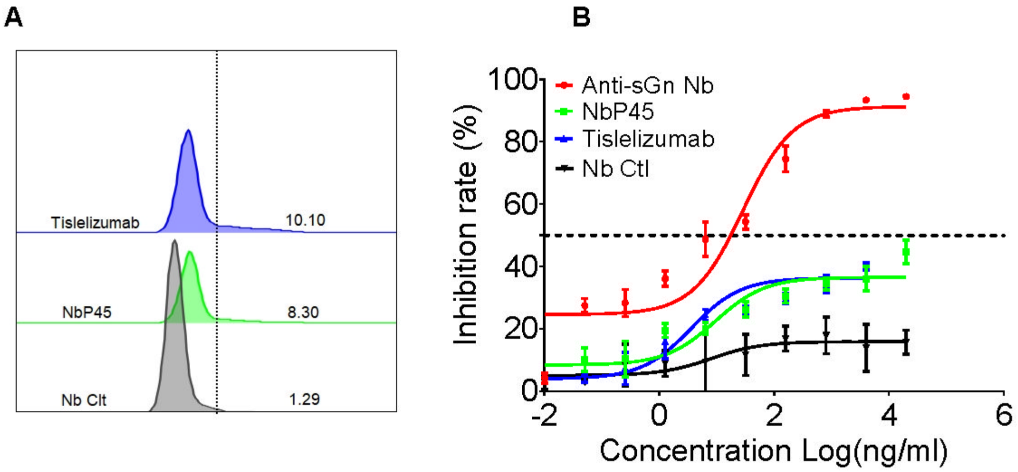

Appendix Figure S3. Characterization of NbP45.

A. Histogram depicting the NbP45 or Tislelizumab binding to PD-1 of THP-1 cells, as evaluated using flow cytometry. Nb Ctl (one published nanobody, Nb15, specific for SARS-CoV-2 S protein) served as negative nanobody control.

B. Neutralization activity of NbP45 (*n* = 3) against live SFTSV infection of PBMCs. Anti-sGn Nb (one nanobody, specific for SFTSV Gn protein), Nb Ctl (one published nanobody, Nb15, specific for SARS-CoV-2 S protein) served as positive and negative nanobody control, respectively.

Data are shown as mean ± SEM.
