# Supplementary material for: Inhibition of SFTSV replication in humanized mice by a subcutaneously administered anti-PD1 nanobody
Source: EMBO Mol Med. 2024 Feb 16;16(3):8. doi: 10.1038/s44321-024-00026-0 (PMC10940662; doi:10.1038/s44321-024-00026-0)
Supplement: Supplementary file 10 — Expanded View Figures [file 44321_2024_26_MOESM10_ESM.pdf]

Expanded View Figures

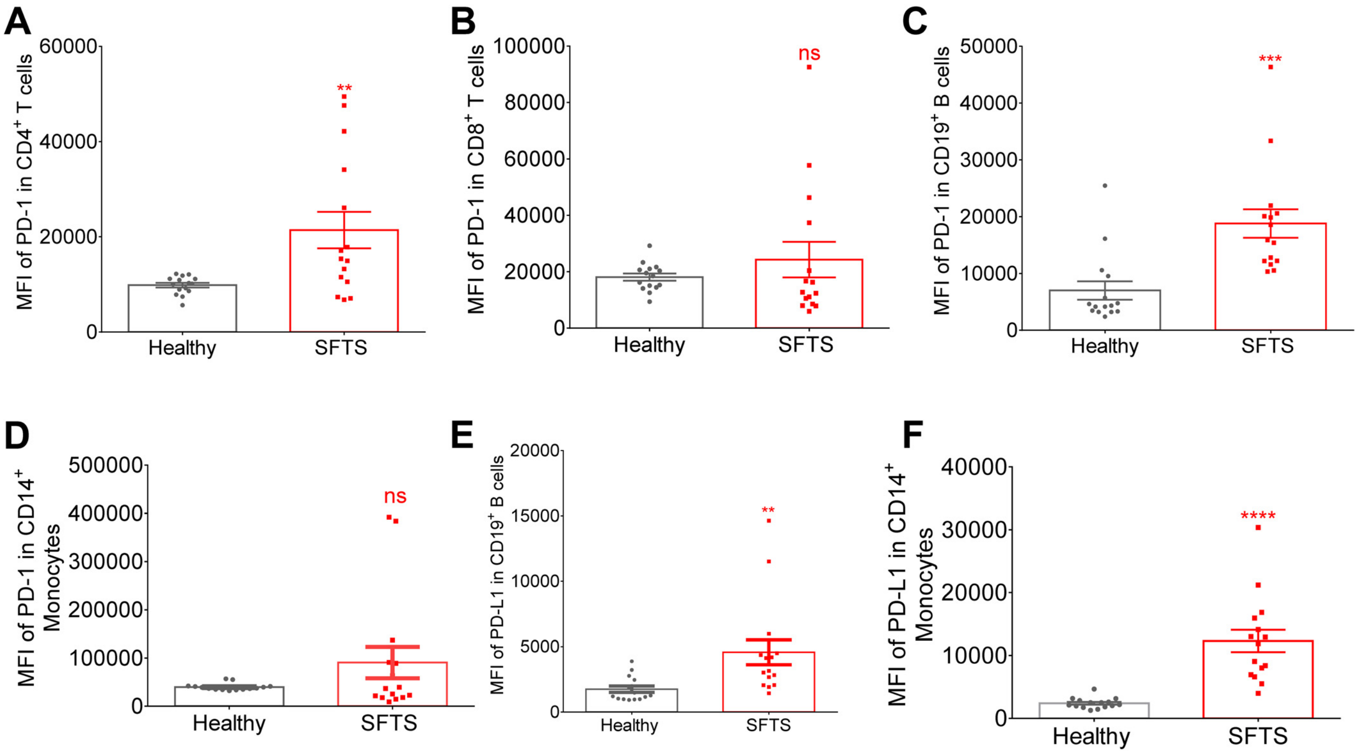

Figure EV1. The expression levels of PD-1/PD-L1 was upregulated in immune cells of SFTS patients.

(**A–D**) The expression levels of PD-1 in $CD4^+$ (**A**) and $CD8^+$ (**B**) T, $CD19^+$ B (**C**) cells and $CD14^+$ monocytes (**D**) was summarized for the SFTS patients ($n = 15$) and the healthy control ($n = 15$). Two-tailed unpaired *t* test was performed to compare SFTS patients with healthy control (ns, no significance; $^{**}P = 0.0058$; $^{***}P = 0.0005$). (**E, F**) The expression levels of PD-L1 in $CD19^+$ B cells (**E**) and $CD14^+$ monocytes (**F**) was summarized for the SFTS patients ($n = 15$) and the healthy control ($n = 15$). Two-tailed unpaired *t* test was performed to compare SFTS patients with healthy control ($^{**}P = 0.0079$; $^{****}P < 0.0001$). Data information: (**A–F**) data are shown as mean ± SEM. ns, no significance; $^{**}P < 0.01$; $^{***}P < 0.001$; $^{****}P < 0.0001$.

© The Author(s)

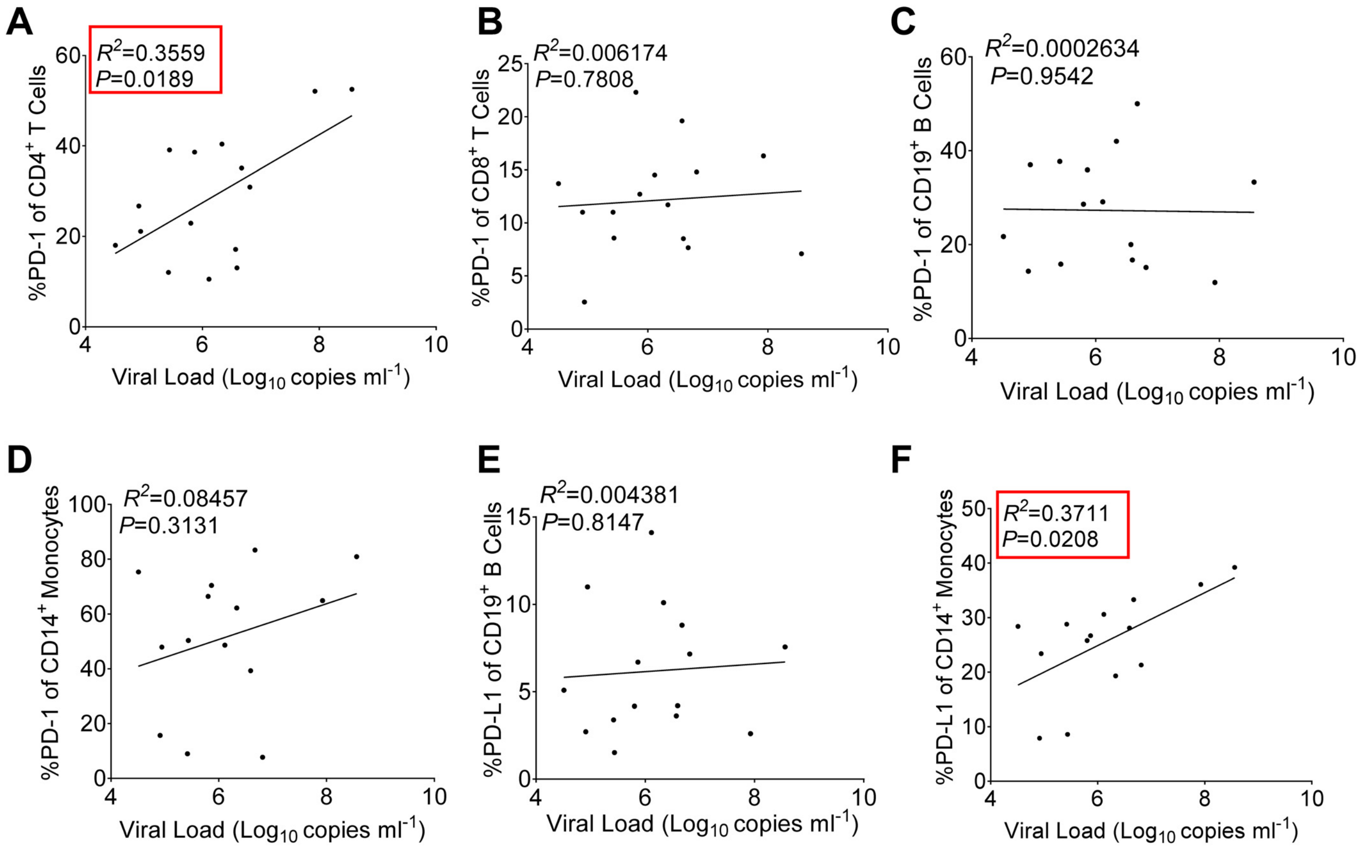

Figure EV2. Correlation between PD-1/PD-L1 expression and serum viral load in SFTS patients.

(**A–D**) Correlation between PD-1 in CD4⁺ (**A**) and CD8⁺ (**B**) T, CD19⁺ B (**C**) cells and CD14⁺ monocytes (**D**) expression and serum viral load in SFTS patients. (**E**, **F**) Correlation between PD-L1 in CD19⁺ B cells (**E**) and CD14⁺ monocytes (**F**) and viral RNA copies in serum viral load in SFTS patients. Data information: Correlation analyses were performed by linear regression using the GraphPad Prism 6.0 program, Pearson's correlation tests were used to measure the strength of association between variables.

 © The Author(s)

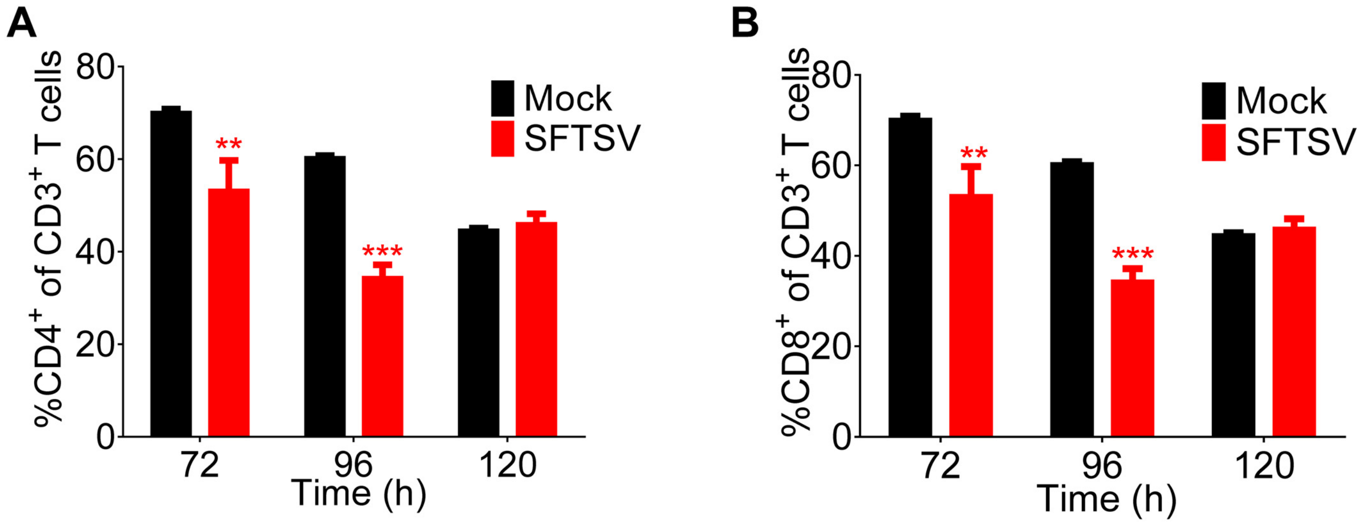

Figure EV3. Kinetics of the T/B lymphocytes during SFTSV-infected PBMC.

(**A**, **B**) The expression of CD4$^+$ (**A**) and CD8$^+$ (**B**) T cells was summarized for the uninfected controls ($n = 3$) and the SFTSV (MOI = 1) infection ($n = 3$) at 72/96/120 h. Two-way ANOVA with Sidak's multiple comparisons test was performed to compare SFTSV infection with uninfected control (**$P = 0.0053$; ***$P = 0.0001$). Data information: Data are shown as mean ± SEM. *ns*, no significance; **$P < 0.01$; ***$P < 0.001$. The *n* means the numbers of sample repeats in one experiment at a same time.

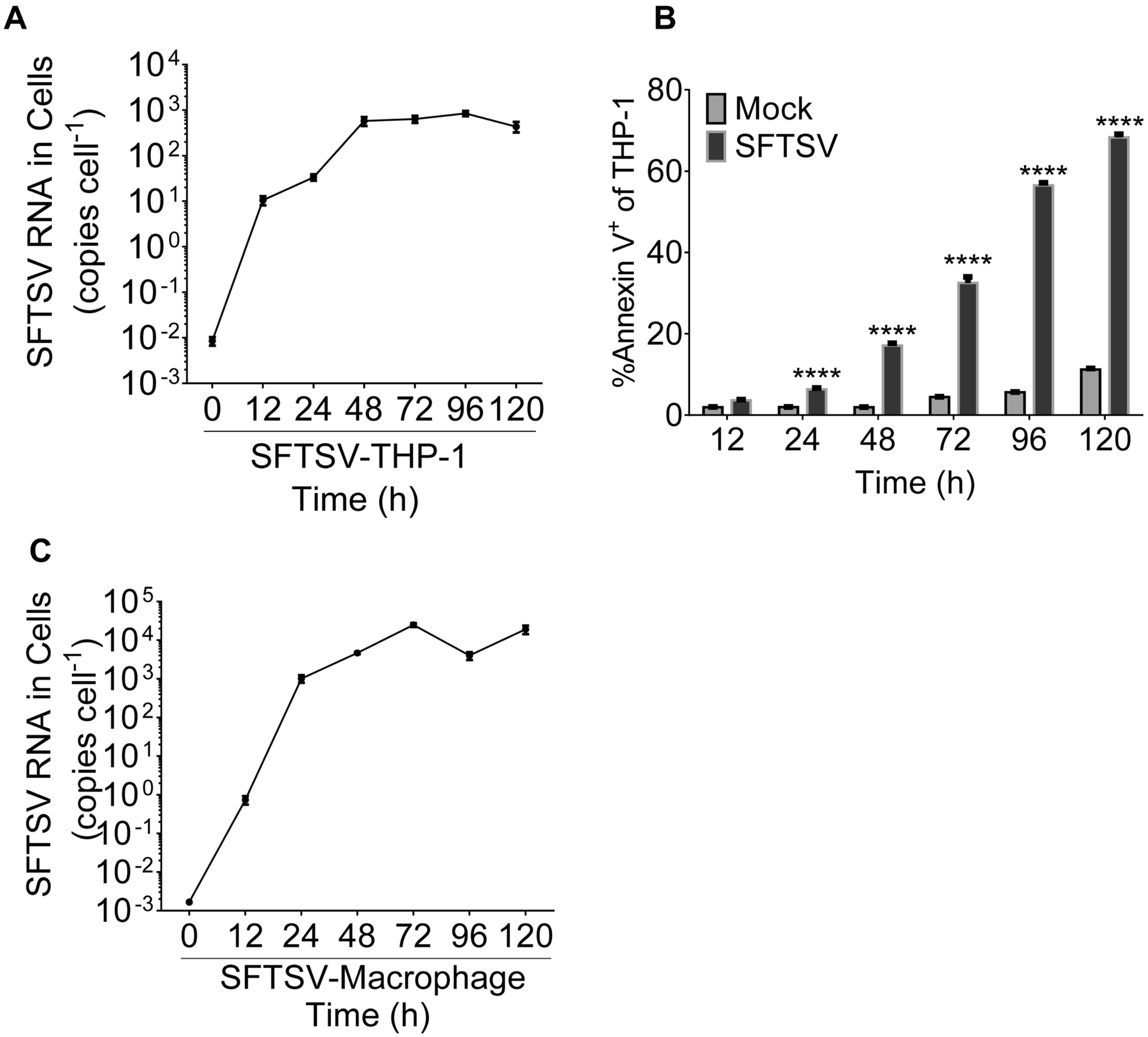

Figure EV4. Kinetics of viral replication in THP-1/Macrophage cells was examined by serial sampling of cells.

(**A**) Kinetics of viral replication in THP-1 cells was examined at SFTSV (MOI = 1) infection (*n* = 3). (**B**) Annexin V^+ expression of THP-1 cells was summarized for the uninfected controls (*n* = 3) and the SFTSV (MOI = 1) infection (*n* = 3). Two-way ANOVA with Sidak's multiple comparisons test was performed to compare SFTSV infection with uninfected control (****$P < 0.0001$). (**C**) Kinetics of viral replication in macrophage cells was examined at SFTSV (MOI = 1) infection (*n* = 3). Data information: Data are shown as mean ± SEM. ****$P < 0.0001$. The *n* means the numbers of sample repeats in one experiment at a same time.

 © The Author(s)

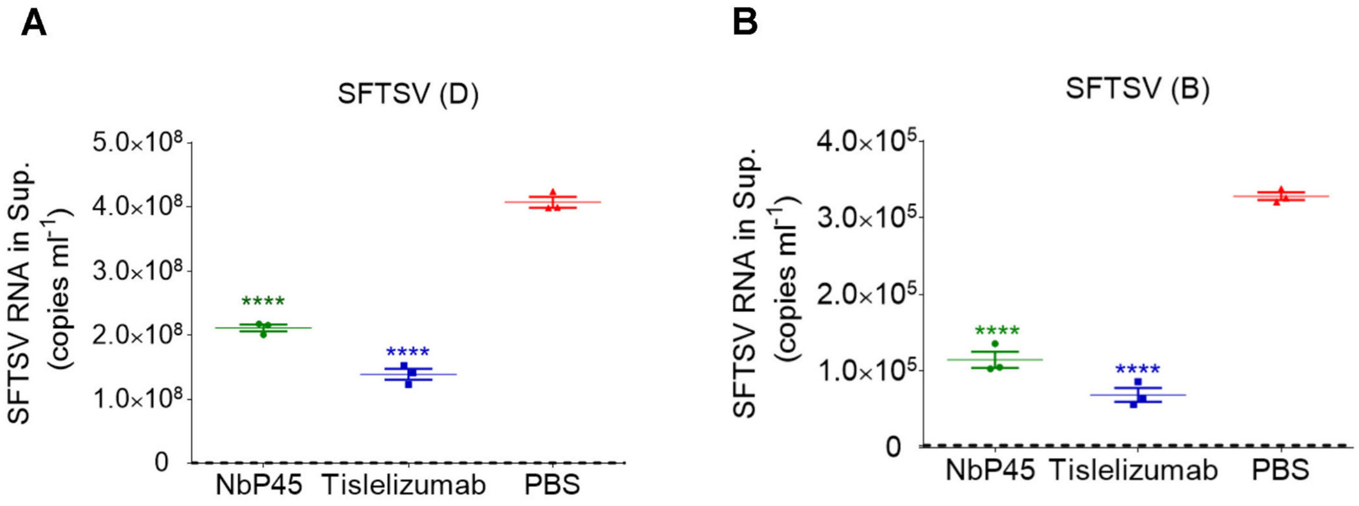

Figure EV5. NbP45 potently inhibited another strain of SFTSV replication.

(**A**, **B**). The inhibition activity of NbP45 ($n = 3$) or Tislelizumab ($n = 3$) against SFTSV of subtype D (**A**) or subtype B (**B**) (MOI = 1) infection PBMCs at 48 hpi. The black dashed line was non-infected control. One-way ANOVA with Tukey's test was performed to compare treatment group with control group (PBS) (****$P < 0.0001$). Data information: Data are shown as mean ± SEM. ****P <0.0001. The n means the numbers of sample repeats in one experiment at a same time.
